# A Hybrid Energy Harvesting Design for On-Body Internet-of-Things (IoT) Networks

**DOI:** 10.3390/s20020407

**Published:** 2020-01-10

**Authors:** Omar A. Saraereh, Amer Alsaraira, Imran Khan, Bong Jun Choi

**Affiliations:** 1 Department of Electrical Engineering, Hashemite University, Zarqa 13133, Jordan; eloas2@hu.edu.jo; 2 College of Engineering and Technology, American University of the Middle East, Kuwait; amer.alsaraira@aum.edu.kw; 3 Department of Electrical Engineering, Engineering University Peshawar, Peshawar 814, Pakistan; ikn.eup121@gmail.com; 4 School of Computer Science and Engineering, Soongsil University, Seoul 06978, Korea

**Keywords:** Internet-of-Things (IoT), energy harvesting, lifecycle, thermoelectric generator (TEG)

## Abstract

The Internet-of-things (IoT) has been gradually paving the way for the pervasive connectivity of wireless networks. Due to the ability to connect a number of devices to the Internet, many applications of IoT networks have recently been proposed. Though these applications range from industrial automation to smart homes, healthcare applications are the most critical. Providing reliable connectivity among wearables and other monitoring devices is one of the major tasks of such healthcare networks. The main source of power for such low-powered IoT devices is the batteries, which have a limited lifetime and need to be replaced or recharged periodically. In order to improve their lifecycle, one of the most promising proposals is to harvest energy from the ambient resources in the environment. For this purpose, we designed an energy harvesting protocol that harvests energy from two ambient energy sources, namely radio frequency (RF) at 2.4 GHz and thermal energy. A rectenna is used to harvest RF energy, while the thermoelectric generator (TEG) is employed to harvest human thermal energy. To verify the proposed design, extensive simulations are performed in Green Castalia, which is a framework that is used with the Castalia simulator in OMNeT++. The results show significant improvements in terms of the harvested energy and lifecycle improvement of IoT devices.

## 1. Introduction

**Figure 1:**
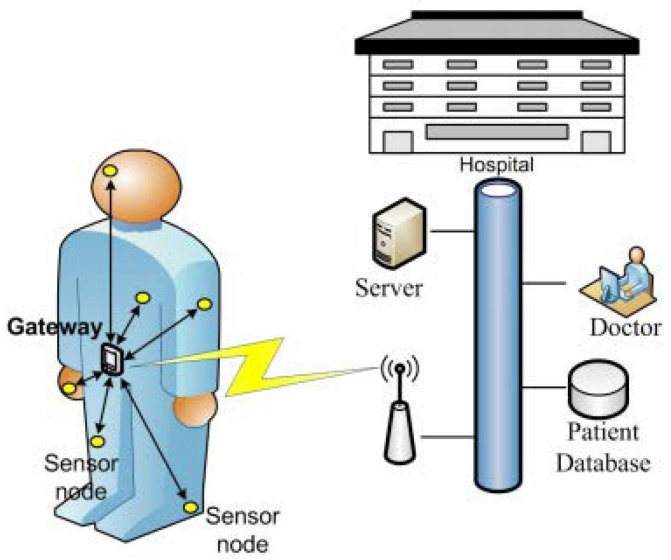
Network illustration of an on-body IoT network. The on-body sensor communicates to a gateway, which then forwards the data to a central database with the hospitals.

In recent years, the demands of low-powered embedded devices and sensors have increased exponentially [1]. Wireless sensors can be used in multiple domains, including medical health monitoring, agriculture, engineering, automobiles, and environment monitoring [2]. In the medical health system, sensors are used to remotely monitor the conditions of patients and transport the patient’s health condition signals to the relevant entity (e.g., doctors, nurses, caring persons, and remote servers). This rapid evolution of connected sensor devices has given birth to the domain of Internet-of-Things (IoT) for healthcare [3], which is illustrated in Figure 1. However, the main problem with IoT sensors is the low-powered nature of these devices. Most often, the IoT sensors are considered useful, until the power is supplied by a battery. Thus, the lifetime of the IoT network remains limited and less scalable, since it highly depends on the energy capacity stored in the battery [4]. Recently, many research efforts have been made to extend the lifetime of IoT sensor networks by improving their energy efficiency and reliability of communication. A node placement algorithm called JR-SPEM [5] was proposed to reduce the energy consumption of sensor networks and to prolong the lifetime of the network. A sleep mode scheduling algorithm called MWCDCT [6] was proposed to increase the lifetime of the IoT sensor network by using a subset of nodes for monitoring a target area and transmitting sensed data to the base station. A routing protocol for energy-harvesting wireless sensor networks called the MC-OMLUalgorithm [7] uses a lifetime utility function to maximize the network lifetime, by balancing the maximum remaining energy and the minimum remaining energy.

There are different limitations for every renewable energy source. For example, solar energy is not available at night. This limits the use of solar-powered wireless devices for indoor scenarios, such as hospitals and smart homes [8]. Therefore, energy harvesting from ambient sources can be a solution to provide a constant energy supply to IoT sensor networks. Commonly available sources of energy are radio frequency (RF) and thermal energy that can be used for generating electrical energy in low-powered IoT devices. Recent studies on the energy harvesting of wireless devices have incorporated a number of aspects of such networks that range from physical layer security [9,10] to cognitive radio networks [11] and non-orthogonal multiple access [12,13]. However, the most focused source of energy harvesting in such works has been on thermal and vibration (mechanical) types of energy harvesters.

### 1.1. Related Work

Liu et al. and Chen et al. [14,15] analyzed the outage probability of body area networks when operating in a cellular band. Due to this operation in a cellular network, the on-body sensors become susceptible to interference from other cellular users. To address this, dynamic resource allocation schemes were proposed in [16]. In this direction, a novel network resource allocation algorithm was proposed to improve the performance of on-body wireless networks in [17]. Similar work was done in [18] where a point-to-point model was investigated, and different wireless body area network protocols were evaluated. To improve the energy efficiency of on-body sensors, Rabby et al. [19] provided a detailed discussion of the source scheduling approach, which offered a novel approach to deal with the high power budget of such networks. In-body implants were studied in [20]. Another work [21] provided a solution for energy harvesting from wide-band vibrations. More specifically, the authors of that work performed experiments and measurements for a piezoelectric converter that showed improvement in overall performance. A novel method was provided to self-sustain the in-body implants to work in a battery-free manner. A relay selection technique was proposed to improve the coverage performance of the on-body network in [22]. A similar approach was adopted in [23], where three different algorithms were proposed for efficient node placement in the network. An energy localization technique for weakly coupled oscillators was proposed in [24]. Energy localization was achieved by mistuning the mass of one of the moving magnets, and the results indicate the benefits of the energy localization phenomenon.

Radio waves from a nearby cellular tower can be used for RF energy harvesting. The receiver has multiple diodes, and the voltage level or received power stream is stored in a supercapacitor. Thermal energy harvesting uses a temperature gradient between the human body and the surrounding environment to produce electricity [25]. Thermal energy can be obtained from the human body using a thermoelectric generator (TEG). More specifically, a TEG converts human heat into direct current (DC) voltage; subsequently, the converted energy is stored in a supercapacitor. The stored energy is then boosted using amplifiers to increase the voltage levels and is then utilized by the sensor node [26]. In this way, an IoT sensor can be made to work in a battery-free manner, thereby increasing the reliability of the network and reducing the maintenance cost. Typically, an energy management policy takes care of aspects such as the energy refill process and the frequency. Due to the random supply of energy in such networks, an efficient energy management policy is key to building a reliable network of nodes [27].

### 1.2. Motivation and Contribution

The aforementioned studies clearly indicate the discrepancy in the existing literature on hybrid energy harvesting techniques for low-powered IoT sensors. Though much work has been done in the domain of RF energy harvesting, very few studies can be found where thermal energy harvesting is employed to improve the lifetime of IoT networks. From this perspective, we anticipate that a juxtaposition of both RF and thermal energy harvesting can provide significant gains in terms of network lifetime. With this motivation, our work provides a unique hybrid energy harvesting design that makes use of both RF and thermal energy in the environment. To evaluate the performance of our proposed framework, extensive simulations have been performed in Green Castalia. The results indicate that using a hybrid energy harvesting method can obtain significant improvements in network lifetime.

The remainder of the paper is organized as follows. Section 2 provides a general discussion on RF and thermal energy harvesting techniques. Section 3 presents the details of the proposed energy harvesting network design. Section 4 provides the details of different modules, while Section 5 describes Green Castalia. Section 6 presents results and a corresponding discussion, and Section 7 concludes the paper. Table 1 lists the acronyms and their definitions.

## 2. Energy Harvesting Techniques

This section provides details of RF and thermal energy harvesting and identifies the key differences between the two techniques. By efficiently utilizing thermal energy, along with RF energy, we anticipate that significant gains can be obtained.

### 2.1. Energy Harvesting

Energy harvesting (also called power harvesting) is a technique through which energy is scavenged from external sources (e.g., solar energy, thermal energy, wind energy, salinity gradients, and kinetic energy) and used by energy-constrained wireless autonomous devices, such as those used in wearable electronics and IoT sensor networks [28]. Energy harvesting technology has been around for many years, as it has evolved from wind turbines, solar panels, and hydroelectric generators. Energy harvesting technology is vital to the future energy-hungry society, due to the fact that the amount of energy that can be harvested is omnipresent, and this process is eco-friendly. Furthermore, the maintenance cost of such systems is not so high. A study from Texas Instruments on the amount of harvested power from various sources shows the potential of energy harvesting [29]. Table 2 highlights some results from this study.

### 2.2. Energy Harvesting Sources

Energy harvesting distribution is established on the basis of the energy used to harvest power. The assorted sources of energy harvesting are photovoltaic cells, solar energy, wind turbine, electromagnetic wave, and mechanical vibration (e.g., piezoelectric) [5]. Table 3 provides a detailed comparison of these techniques.

#### 2.2.1. RF Energy Harvesting

**Figure 2:**
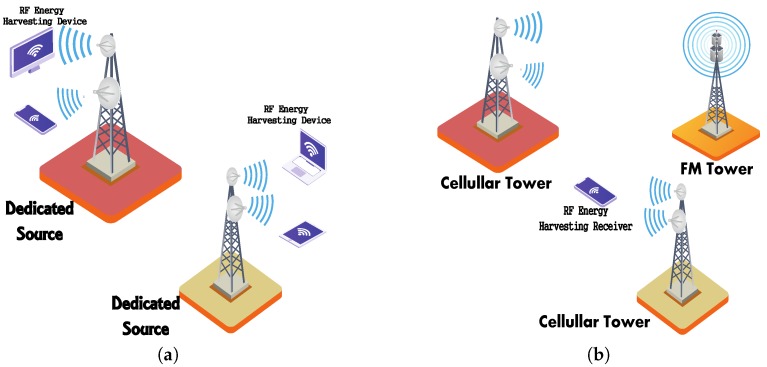
Illustration of dedicated and ambient energy harvesting. (**a**) Dedicated Source; (**b**) Cellular Tower.

Currently, RF transmission from hundreds of thousands of radio transmitters is eminent around the world, including a mobile base station, a cellular phone, and television broadcasting terminals. The capability of harvesting RF energy from surrounding or dedicated sources facilitates the charging of mobile devices and has cumulative benefits on product design, adoption, and reliability [31]. Devices are free of wires, components, and connectors to facilitate battery replacement, and the capability and portability of low-power devices can be extended. Figure 2a,b illustrates dedicated RF energy harvesting and ambient RF energy harvesting, respectively. More specifically, dedicated energy harvesting refers to a harvesting scheme where RF sources are dedicated to providing energy to a particular IoT sensor. This can be performed by initially synchronizing the source and the receivers. However, ambient energy harvesting is more complex, since the receiver has to harvest energy without cooperation from the source. This may result in variable energy harvesting gains over a long period of time, due to the dynamism of wireless networks. At a close range of flat power transmitters, RF energy can be used to charge many devices, together with wearable medical sensors, a global positing system (GPS) module, and end-user electronics, such as a wireless headset and an e-book reader. In a highly populated urban area, electromagnetic waves are celestially available over a wide spectrum of frequencies. If radio waves can be effectively and readily harvested, they can provide a universally available source of energy. The power consumption of electronic components is continuously decreasing, while the efficiency of RF energy harvesting devices is increasing. Therefore, the growing demand for the wireless charging of IoT devices can be met using RF energy harvesting.

#### 2.2.2. Thermal Energy Harvesting

An energy harvesting system that uses input heat as a source to convert it to electrical energy is called a thermoelectric potential harvester. Thermal energy produced by the human body from walking, breathing, or any other body movement can be used as a source of thermal energy harvesting. This thermal energy can be converted into DC voltage by a TEG. Here, the energy conversion efficiency of thermoelectric harvesters depends on the temperature drop across the TEG and on the thermoelectric material exhibiting the conversion.

The TEG is considered a popular harvesting resource, compared to the auxiliary harvesting resource, because of its compact design. With the constant availability of resources and due to its simple design, energy harvesting is more productive and direct. There are also no moving parts in the system design. The disadvantage of the thermoelectric harvester is that the amount of harvested energy is low, as compared to other types of energy harvesters.

## 3. Proposed Energy Harvesting Design for On-Body Networks

The demand for IoT sensor networks in healthcare applications is dramatically increasing. In such networks, sensor signal data received from the patients are processed and communicated to the healthcare personnel (i.e., doctor, nurses, etc.), who accordingly make decisions. Therefore, the operation of the on-body IoT network relies on the lifecycle of the sensors [32]. Localizing the gateway that can be used for communication between patient and healthcare units is an important issue [33]. Healthcare systems can either be a standalone system, such as an on-body IoT network, or an element of a global health care system [34], which is connected directly or indirectly to the Internet at all times. The medical staff can collect and use the data to timely acquire the status of patients, e.g., arrhythmia events and abnormal electrocardiogram (ECG) signals, for correct medical treatment. In addition, physiological data accumulated over a long time can be provided to medical practitioners to assist in diagnosis and treatment. An on-body IoT network equipped with tiny sensors is an effective means of providing important signals from patients to medical practitioners for both monitoring and in-depth analysis.

**Figure 3:**
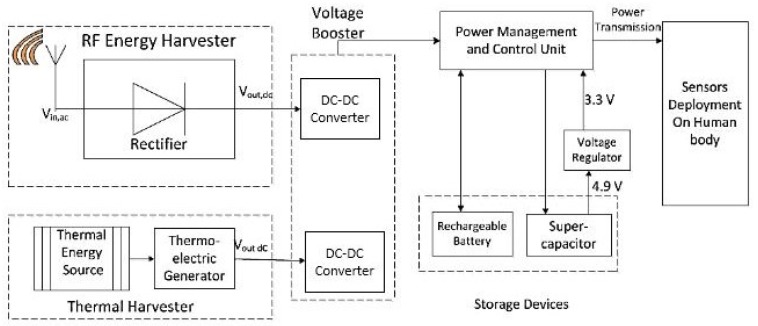
Proposed hybrid energy harvesting receiver design.

The most common topology of an on-body IoT network is the star-topology, in which a leading sensor node collects and notes sensing information. The communication range of sensor nodes used in the on-body IoT network is within a few meters. The power consumption of these networks is critical for the proper treatment of patients. Figure 3 shows a block diagram of our proposed receiver design. In the subsequent subsections, we detail different components of the proposed energy harvesting receiver design.

### 3.1. Design of Thermal Energy Harvesting

**Figure 4:**
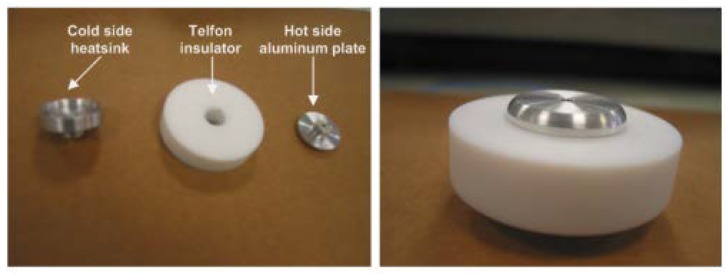
Thermal energy harvesting hardware components.

The warmness of an object can be used as a substantial source of energy for powering sensor nodes in an on-body IoT network. The chunk of energy during the process of metabolism (1 Met = 58.15 W/m${}^{2}$) relies on the measure of muscular activity [35,36]. Given the amount of thermal energy input, an appropriate design of thermal energy harvesting (TEH) architecture with a thermoelectric generator (TEG) can be constructed. Figure 4 shows a thermoelectric generator that is made from aluminum and Teflon consisting of two plates. Aluminum plates in TEG have shown to perform better than the conventional ceramic plates [37,38]. One is a hot plate that is fabricated with aluminum to gather heat as quickly as possible. The other is a cold plate that is also fabricated with aluminum to be used as a heat diffuser. The insulator is made of Teflon, which is put between the two plates to adequately minimize relocation and the emission of heat from the cold plate and hot plate. Moreover, it helps to avoid overheating, which is not desirable, because it minimizes the thermal gradient between the two plates.

### 3.2. RF Energy Harvesting Structure With Rectenna

The harvested energy from RF sources provides more flexible installation constraints, as compared to other types of energy sources. For example, the non-contact charging feature makes wireless power transfer the most popular method of powering implantable biomedical devices [39,40]. An RF antenna intercepts and converts electromagnetic waves to voltages. The RF harvester can be designed as a voltage source that is in series with corresponding resistances. Generally, matching impedance is required at the antenna interface, so that a small amount of power in reverse, as well as the ultimate input power, also known as the available power, can be computed. The harvester takes action on the received power and charges the storage unit as long as the input power is accessible [41].

Because the RF signal is used for wireless communication, the amount of energy harvested is limited and subject to long-term fluctuations. Still, RF energy harvesting is more consistent in terms of energy generation than any other harvesting source.

### 3.3. Power Management

The power management structure in thermal energy harvesting and RF energy harvesting design consists of an energy storage unit. The power harvested by the TEG and the rectenna is stored in the capacitor to be later supplied to sensor loads. While the capacitor is being charged, both switches are opened to separate the TEG. When the voltage level of the capacitor approaches a predefined threshold of 4.9 V, the switch Q1 is closed, and this also triggers the other switch Q2. The energy stored in the capacitor is discharged, which brings down the input voltage from 4.9 to 3.3 V, and power is supplied to sensor loads.

### 3.4. Fall Detection

The proposed on-body IoT network implements a (human) fall detection system, which is a popular application for on-body IoT networks. Sensor nodes are mounted on the body to notify of fall events. Whenever a fall is detected, the resulting signal data are forwarded via wireless communication to a destination, e.g., a base station, which pre-processes the signal and forwards it to the appropriate medical practitioner, who then diagnoses the condition of the fallen patient. A fall detection event is monitored using accelerometers. As a requirement of the given application, it is assumed that the accelerometer is able to differentiate between an upright standing posture and a fallen posture, using its internal functions and output signals that represent different postures [42].

### 3.5. System Integration

The realization of the wearable health monitoring system mainly depends on how well the system is integrated. Coherent integration tailored for individual health monitoring will promote the user’s acceptance and consent, while integration of analysis and action into electronic medical evidence and a health monitoring system will improve the diagnosis by medical professionals. We consider that a group of intelligent sensors is linked to an on-body IoT network that communicates with a server directly, or via an Internet gateway. The sensor data is then saved in the electronic medical record (EMR) via the Internet. Sensors may not afford enough memory capacity and processing power to provide real-time data processing for large health monitoring applications. A sensor platform initially stores unacknowledged messages in local memory. This can provide significant advantages over the primary generation of telemetry systems with the transportation of raw signals.

### 3.6. Control Unit

A control unit is used to control and manage power generated by the energy harvested source. Electrical DC power harvested from the sources is gathered in the supercapacitor until an acceptable level is reached to power the loads. The procedure of storing and delivering energy is composed through the supply circuit with two Metal–Oxide–Semiconductor Field-Effect Transisto (MOSFET )switches. During the time when the capacitor is being charged, both switches are opened to remove the source of harvested energy from the load. In this case, power is supplied from the battery to the load. After the capacitor is fully charged, both switches are closed, and power is supplied to the load from the supercapacitor. The energy stored in the storage unit needs to be controlled. The energy is stored in the capacitor is discharged to bring down the input voltage from 4.9 to 3.3 V. A voltage regulator is used to step down the voltage to 3.3 V and feed this voltage to the power controller. During the time when the capacitor is discharged, the power controller transmits power from a nickel–metal hydride (NiMH) battery source. When the capacitor is fully charged, the battery source is disconnected, and power is supplied from the supercapacitor.

## 4. Modules and Associated Components

In order to replicate the aforementioned receiver design for simulations, we introduce the concept of modules. In particular, the modules represent the boxes in the network having a predefined set of inputs and outputs. Communication between modules is carried out by exchanging messages. A module accepts messages from other modules and runs a piece of code. Messages are called packets or frames within a network. A sensor node is operated with a power supply and is controlled by a control and management unit.

### 4.1. Battery Module

The battery is a crucial component of the on-body IoT network, as it is the only source of power for all the hardware components in the sensor network. The energy level of the battery decreases as energy is consumed by the components. We assume that the power consumption of sensors can be accurately estimated; hence, the battery lifetime (*T*) can be calculated as $T=P/\sum I_{j}$, where *T* is the lifetime of the battery, *P* is the total power of the battery, and $I_{j}$ is the current drawn by the *j*-th sensor node. Obviously, because the capacity of the battery is finite, the battery needs to be replaced when its lifetime expires or is about to expire. Energy harvesting modules can be introduced to mitigate this problem.

### 4.2. RF Energy Harvesting Module

RF energy is available from the ambient environment (2.4 GHz) and can be converted into electrical energy by means of the rectenna. A rectenna is a special antenna developed to convert RF energy to DC electrical energy. Due to its unpredictable behavior, energy harvested from ambient sources is an opportunistic process that requires some adaptivity and a sophisticated design at both the circuit and system levels. The energy harvesting module generally consists of a sensor interface, rectenna, storage unit, transceiver, and power management unit. The conversion from RF to DC using the rectenna yields energy loss that is measured in terms of power conversion efficiency (PCE), which is the ratio of converted DC power to input RF power. It has significant meaning in the overall performance of the harvesting unit. As expected, low-power RF signal harvesting is more challenging than high-power RF signal harvesting. For example, a high antenna gain (e.g., 6) is needed, as the RF energy is sometimes below −20 dB, due to fading and shadowing. When a low-power signal is rectified, it produces a low-voltage DC signal. Therefore, a voltage adapter (DC–DC converter) can be used to adapt its power, neglecting the converter inner losses.

### 4.3. Thermal Energy Harvesting Module

The heat of the human body is used as a source of electric energy to power up sensor nodes in an on-body IoT network. The extent of energy liberated by the human body depends on the subject’s activity. In order to use this released energy as electrical energy, this heat energy needs to be converted into electrical energy. The thermoelectric generator is used to convert thermal heat into electrical energy. Since the TEG output voltage is directly proportional to the temperature variation ΔT across the TEG [42], the output voltage of the TEG is not constant, as the temperature across the TEG is unstable.

### 4.4. Hybrid Energy Harvesting Module

A resultant energy harvesting module is designed that integrates the RF energy and thermal energy exhaust by the human body. Both of the proposed energy harvesters receive input energy from the surroundings and convert it to DC voltage. The harvested energy is too small to be directly applied to the load. A voltage booster is used to increase the low voltage level to a sufficient level and is controlled by the power management and control unit. The control unit tool decision is based on the amount of energy harvested, the remaining energy of the battery, and the remaining energy of the supercapacitor.

The voltage regulator is used to step down the voltage from 4.9 to 3.3 V for the smooth operation of sensor nodes. The remaining energy level of each sensor is monitored, and for communication purposes, a sensor is selected randomly that has the highest energy level among all sensors. If the energy level of the currently selected sensor is decreased, then the energy level of each sensor is scanned again, and the highest remaining level of energy sensor is selected as a gateway. Based on the data received, the action is initiated accordingly.

### 4.5. Storage Unit Module

Sensors do not have any permanent source of energy. The battery is the only source of energy. The lifetime of the wireless sensor network is limited, as the energy stored in the battery is drawn by different components. The rechargeable battery and a supercapacitor are used to store harvested energy. The capacity of the supercapacitor to store voltage is 4.9 V. The storage unit is designed to collect flat level input energy to a long-haul storage material, which constitutes a sufficient amount of energy for sensing and communication. The supercapacitor has a leakage current and equivalent series resistance that are as small as possible. The supercapacitor stores energy until it reaches its maximum storage capacity, and then it releases energy to the load. A voltage comparator is used to indicate whether the voltage level is high. This is needed to ensure that the energy stored in the storage capacitor is accumulated until a certain voltage is reached. During the time when the capacitor is being charged, power is supplied to the load from the battery. When the capacitor is fully charged, it supplies power to the load, and battery power is saved. In this work, a NiMH rechargeable battery is used [43]. The NiMH battery discharges at the level of 1.5 V.

### 4.6. Load Unit Module

Different sensors are used for different purposes and require different amounts of power to operate. For this purpose, a mesh topology is used to interconnect all sensor nodes. Nodes in the on-body IoT network have a micro-controller/processor. The processor requires different levels of energy in active, idle, and sleep states. The energy level of each sensor node is calculated and compared with the energy level of other sensor nodes. A sensor node with high power among all sensor nodes is selected as a gateway that collects data from other sensor nodes and communicates with loads and servers.

### 4.7. Communication Module

The communication module provides connections among sensor nodes. A collision can occur if two or more sensor nodes simultaneously send their data over the wireless channel. In order to avoid a collision, we need to know about the state of the channel at the medium access control (MAC) layer, i.e., whether the station is busy or unproductive for a certain amount of time. The hybrid communication protocol, carrier sense multiple access collision avoidance (CSMA/CA), is used with time division multiple accesses (TDMA). The consumption of CSMA/CA is very low. It is not preferred in on-body IoT networks for avoiding collisions. Therefore, CSMA/CA is suited for this wireless sensor network. In addition, TDMA assigns a time slot for all sensor nodes over the wireless network. The TDMA scheme requires a high degree of time synchronization.

## 5. Simulation Platform

This section provides details of the simulation platform and the performance evaluation method. To simulate the aforementioned receiver design, Green Castalia has been used, which is an extension of the prominent Castalia simulator [44]. The Green Castalia simulator allows for the modeling and simulating networks of on-body sensor devices with energy-harvesting capabilities. The Green Castalia framework is integrated with the Castalia simulator, stretching it with a flexible framework to reproduce networks of on-body devices with heterogeneous energy harvesting and storage potential. Furthermore, it employs a multi-source harvesting architecture which makes use of both RF and thermal energy harvesting techniques. The resource manager module is responsible for energy harvesting and management. It is also worth mentioning that simulations in Green Castalia are carried out by designing different energy modules for different purposes and integrating all in a cohesive manner.

In the Castalia framework, the energy subsystem is a compound module, is responsible for energy management, and occupies energy-specific parameters that contain baseline power consumption and the initial energy budget of the mote. The remaining energy from the sensor node is also recorded for future use by sending routine updates. The energy subsystem implements the basis for energy harvesting even at a sensor node level, by managing and integrating sub-modules that perform energy harvesting.

An energy subsystem module contains three sub-modules:

- **Energy Harvester:** This module models energy harvesting operation and handles the comparable device.
- **Energy Storage:** This module produces energy repository devices, such as supercapacitors and batteries, which are either disposable or rechargeable.
- **Energy Manager:** This module provides the control logic for repository application and charging.

A brief description of these energy sub-modules is given below:

### 5.1. Energy Harvester

The energy harvester module is associated with a sensor node of the physical harvesting device. The default interface of the energy harvester permits the specification of the source that is attaching to it, as well as the highest power it is generating. It also indicates a time-stamped file that indicates the time-to-time efficiency of the harvesting devices. In addition, it allows for building time-varying issues such as shadows, decreases in efficiency because of dust, briefly, obstruction, and any change that occurs in the harvester direction. The availability of energy of the harvesting device also traces with the help of parameters described by efficient inter-device communications [45].

### 5.2. Energy Storage

The energy that is supplied to the sensor node by a storage device is represented by energy storage. Basic parameters, such as charging, discharging, and high rated potential, are the interfaces of the energy storage module. Both storage devices have the function of charging that is again labeled by the energy manager at the time when excess energy is available. The function of self-discharging is also implemented in both storage devices. The purpose of this function is to identify the effect of leakage and of the self-discharging of both devices.

### 5.3. Energy Manager

The core of the energy subsystem is the energy manager module. This performs all control tasks, such as storage device usage, the harvester, and storage device simulation for the flow of energy to the desired load and the energy store in a storage device that is accumulated by harvesters [46]. Energy wasted by storage devices is also identified due to their characteristics, such as the efficiency of charging and discharging, the automatic discharge, and the finite capacity, which result in losses of energy, due to the process of harvesting. A function known as energy update causes the energy to update and trigger the hardware component’s state as a result of a change in energy consumed by the devices. As the energy update function is invoked, the energy manager module calculates the net power consumed by the sensor node, considering ongoing power utilization and the rate of harvesting. If the current power that is harvested is negative, then fat energy is utilized to recharge both storage devices or one of them. If it is not so, then power is supplied to the sensor node from the storage device, and they start discharging.

## 6. Results and Discussion

This section deals with the obtained simulation results and presents a corresponding discussion. In the simulations, a network of 36 sensor nodes is built, and each node has different interactive modules, as discussed in the previous section. The initial energy and maximum energy of the node are the same. The initial energy is the energy of the node when it is in an active state. Each node’s inactive state sends or receives packets to other nodes or sinks. The number of packets sent and received by each node is different, depending on the nature of the operation of the node and type of data.

**Figure 5:**
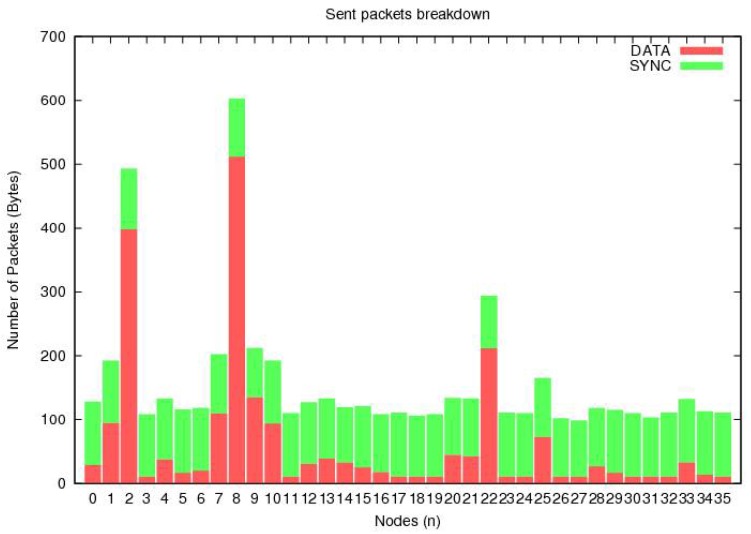
The number of packets as a function of total number of IoT nodes in the network.

Figure 5 shows the number of packets sent by each node. Here, the horizontal axis indicates the number of sensor nodes, while the vertical axis shows the number of packets sent by each node. It is evident that all of the nodes sent packets in a random manner, which is very close to the practical communication conditions. The sent packets can be divided into two categories, i.e., synchronization packets and data packets.

**Figure 6:**
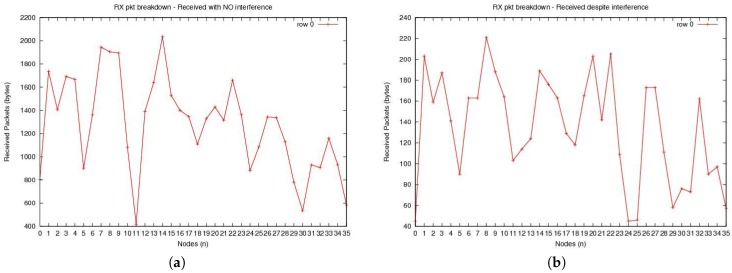
The number of packets received (**a**) without interference, and (**b**) with interference.

Figure 6 demonstrates the number of received packets with and without interference. Due to the use of collision avoidance protocols, Figure 6a shows that the number of received packets with no interference is greater. In contrast, Figure 6b shows that the packets with interference are few in number. This shows the communication efficiency of the IoT network setup.

**Figure 7:**
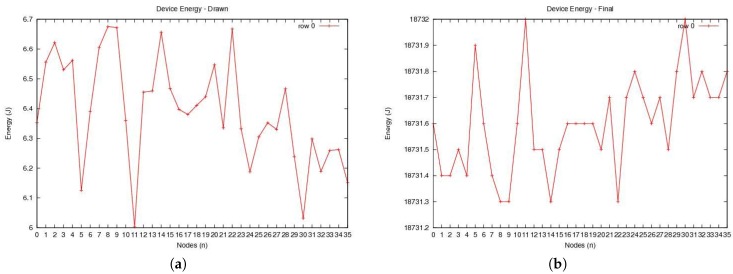
Energy profile: (**a**) the amount of energy drawn and (**b**) the remaining amount of energy.

Figure 7a assumes that the initial energy level of all sensor devices is the same. The figure shows that different sensor devices need different energy levels to operate. It also shows that the amount of energy spent by each node to perform its operation is different. In practice, some sensor nodes may require more energy than others to complete their tasks. As a result, the amount of energy remaining after drawing energy to complete the task by each sensor node may be different. Thus, Figure 7b shows the remaining energy for the sensor nodes.

**Figure 8:**
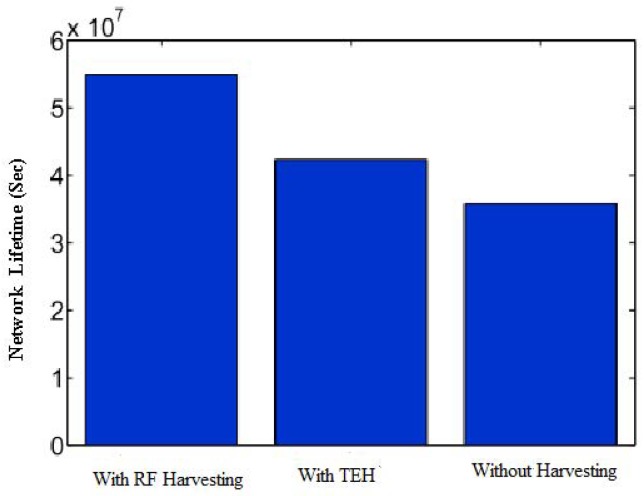
Comparative analysis of RF and thermal energy harvesting. TEH: Thermoelectric Harvesting.

Figure 8 compares the network lifetime using RF energy harvesting, using thermal energy harvesting, and without using an energy harvesting protocol. Here, the network lifetime without energy harvesting refers to the communication paradigm where no energy harvesting is performed. Since the network feasibility is dependent on the proper working of all the IoT devices in the network, the network lifetime can be referred to as the time when the very first IoT device depletes its energy in the network. It is noticeable that network lifetime is increased by 24 and 36% by using thermal and RF energy harvesting protocol, as compared to the case without using any energy harvesting protocol. Since the sensor network is dependent on all the devices, the depletion of energy at any device impacts the operation of the entire network. Thus, all sensor nodes are operating at maximum energy before the first node becomes unavailable. When the first node energy is depleted, then all other nodes have low remaining energy levels to sustain the network operations.

**Figure 9:**
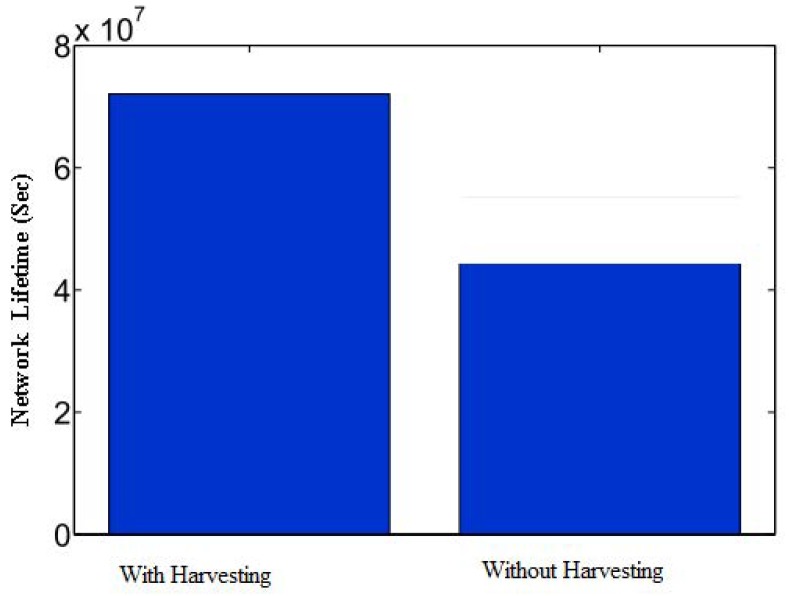
Comparison of hybrid energy harvesting design with and without energy harvesting.

Figure 9 investigates the impact of the energy harvesting mechanism on lifetime maximization. In particular, we calculate the lifetime of an on-body IoT network by using a hybrid energy harvesting solution, in which energy is harvested for 60 s. In the harvested network, nodes harvest energy for 60 s, and forward or receive data in its cyclic duration. The bars in the figure indicate that the lifecycle of the network is increased significantly in a hybrid energy harvesting network, as compared to without an energy harvesting network.

## 7. Conclusions

The importance of on-body IoT networks for providing effective healthcare services cannot be overstated. In this context, this paper has presented a hybrid energy harvester to improve upon the network lifetime of IoT healthcare devices. The considered device model makes use of both the RF energy harvester and the thermal energy harvester to increase the lifetime of devices. In general, it is noted that PCE at 2.4 GHz is about 80%, and the energy harvest is about 0.740 J. The thermal energy harvest at a heat gradient of 15 ${}^{{}^{\circ}}$C is about 0.530 J. Due to the fact that the energy harvested by these two sources can still be improved, the proposed setup employs a supercapacitor to store this energy at a sufficient level to power IoT devices. In general, it has been found that the network lifetime is increased by 24% in the hybrid energy harvesting network, as compared with a network without any energy harvesting. Future studies can build on this work by incorporating other sources of energy harvesting.

## Figures and Tables

**Table 1:** Acronyms and their definitions used in this work.

Acronym	Definitions
RF	Radio Frequency
ine DC	Direct Current
ine GPS	Global Positioning System
ine TEG	Thermoelectric Generator
TEH	Thermoelectric Harvesting
EMR	Electronic Medical Record
ECG	Electrocardiogram
MOSFET	Metal–Oxide–Semiconductor Field-Effect Transistor
NiMH	Nickel-Metal Hydride
JFET	Junction Field-Effect Transistor
MAC	Medium Access Control
ine CSMA/CA	Carrier Sense Multiple Access Collision Avoidance
TDMA	Time Division Multiple Access
PCE	Power Conversion Efficiency
5G	Fifth Generation
GSM	Global System for Mobile Communications
WiFi	Wireless Fidelity
FM	Frequency Modulation
MWCDCT	Minimum Weight Coverage and Data Collection Tree
MC-OMLU	Multi-Commodity Online Maximum Lifetime Utility
IoT	Internet-of-Things
OMNeT++	Objective Modular Network Testbed in C++

**Table 2:** Energy harvesting estimates by Texas Instruments [30].

Energy Source	Harvested Power (μW/cm${}^{2}$)
**Vibration/Motion**
Human	4
Industry	100
**Temperature Difference**
Human	25
Industry	1–10
**Light**
Indoor	10
Outdoor	10,000
**Radio Frequency**
GSM	0.1
Wi-Fi	1

**Table 3:** Comparison of various energy harvesting techniques.

Type	Source of Availability	Energy Conversion Efficiency	Feasibility on Micro-scale Application
**Wind**	Depends on weather	Varies depending on source	Not suitable as wind speed is not consistent
**Heat**	Always available for on-body application	Low	Considerable with power management circuit under specific temperature range
**Solar**	Maximum 6 hours of peak irradiance daily	Maximum during the peak irradiance	Considerable with charge controller
**Hydro**	Available when there is a high-pressure water source	Depends on water pressure	Considerable only under specific flow rate of water
**Kinetic**	Available when there is movement and vibration	Depends on the motion	Very small energy and not suitable for many applications
